# Deep Learning Fast Screening Approach on Cytological Whole Slides for Thyroid Cancer Diagnosis

**DOI:** 10.3390/cancers13153891

**Published:** 2021-08-02

**Authors:** Yi-Jia Lin, Tai-Kuang Chao, Muhammad-Adil Khalil, Yu-Ching Lee, Ding-Zhi Hong, Jia-Jhen Wu, Ching-Wei Wang

**Affiliations:** 1 Department of Pathology, Tri-Service General Hospital, Taipei 11490, Taiwan; B93401052@ntu.edu.tw (Y.-J.L.); chaotai.kuang@msa.hinet.net (T.-K.C.); 2 Institute of Pathology and Parasitology, National Defense Medical Center, Taipei 11490, Taiwan; 608610017@mail.ndmctsgh.edu.tw; 3 Graduate Institute of Biomedical Engineering, National Taiwan University of Science and Technology, Taipei 106335, Taiwan; m10823801@mail.ntust.edu.tw (M.-A.K.); m10923110@gapps.ntust.edu.tw (D.-Z.H.); 4 Graduate Institute of Applied Science and Technology, National Taiwan University of Science and Technology, Taipei 106335, Taiwan; D10522201@mail.ntust.edu.tw

**Keywords:** thyroid cancer diagnosis, thyroid fine needle aspiration, ThinPrep, whole slide image, deep learning, cytology

## Abstract

**Simple Summary:**

Papillary thyroid carcinoma is the most common type of thyroid cancer and could be cured if diagnosed and treated early. In clinical practice, the primary method for determining diagnosis of papillary thyroid carcinoma is manual visual inspection of cytopathology slides, which is difficult, time consuming and subjective with a high inter-observer variability and sometimes causes suboptimal patient management due to false-positive and false-negative results. This study presents a fast, fully automatic and efficient deep learning framework for fast screening of cytological slides for thyroid cancer diagnosis. We confirmed the robustness and effectiveness of the proposed method based on evaluation results from two different types of slides: thyroid fine needle aspiration smears and ThinPrep slides.

**Abstract:**

Thyroid cancer is the most common cancer in the endocrine system, and papillary thyroid carcinoma (PTC) is the most prevalent type of thyroid cancer, accounting for 70 to 80% of all thyroid cancer cases. In clinical practice, visual inspection of cytopathological slides is an essential initial method used by the pathologist to diagnose PTC. Manual visual assessment of the whole slide images is difficult, time consuming, and subjective, with a high inter-observer variability, which can sometimes lead to suboptimal patient management due to false-positive and false-negative. In this study, we present a fully automatic, efficient, and fast deep learning framework for fast screening of papanicolaou-stained thyroid fine needle aspiration (FNA) and ThinPrep (TP) cytological slides. To the authors’ best of knowledge, this work is the first study to build an automated deep learning framework for identification of PTC from both FNA and TP slides. The proposed deep learning framework is evaluated on a dataset of 131 WSIs, and the results show that the proposed method achieves an accuracy of 99%, precision of 85%, recall of 94% and F1-score of 87% in segmentation of PTC in FNA slides and an accuracy of 99%, precision of 97%, recall of 98%, F1-score of 98%, and Jaccard-Index of 96% in TP slides. In addition, the proposed method significantly outperforms the two state-of-the-art deep learning methods, i.e., U-Net and SegNet, in terms of accuracy, recall, F1-score, and Jaccard-Index (p<0.001). Furthermore, for run-time analysis, the proposed fast screening method takes 0.4 min to process a WSI and is 7.8 times faster than U-Net and 9.1 times faster than SegNet, respectively.

## 1. Introduction

**Figure 1:**
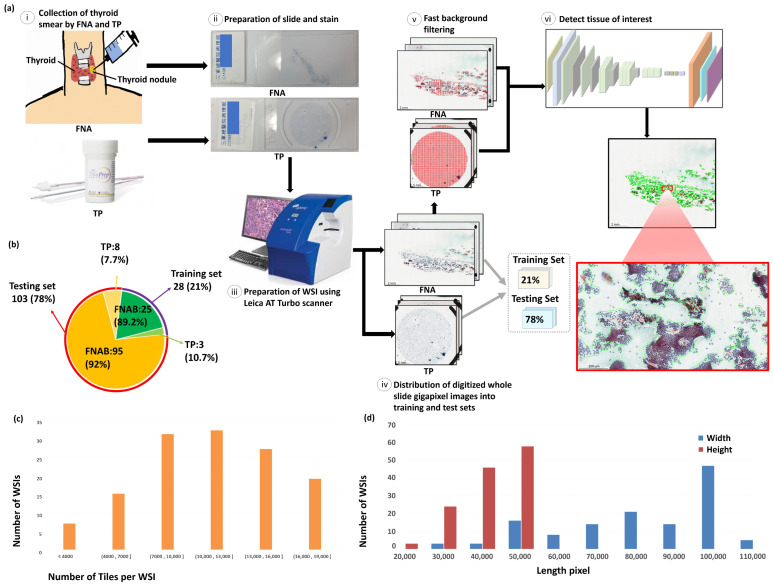
Illustration of the proposed framework structure and the dataset information. (**a**) The proposed framework structure. (**i**) Collection of thyroid smear samples of patients through FNA and TP; (**ii**) preparation of thyroid FNA and TP slides with papanicolaou’s staining; (**iii**) digitalization of cytological slides at 20× objective magnification using Leica AT Turbo scanner; (**iv**) distribution of digitized gigapixel WSIs into separate training set (21%) and testing set (79%); (**v**) processing of WSIs with fast background filtering; (**vi**) identification of PTC tissues of individual WSIs using the proposed deep learning model in seconds. (**b**) Distribution of thyroid FNA and TP cytological slides for training and testing. (**c**) Distribution of tile numbers per WSI. (**d**) Size distribution of the WSIs with width and height as blue and red, respectively.

Thyroid cancer is the most prevalent cancer in the endocrine system and accounts for the majority of head and neck cancer cases [1]. Thyroid cancer has been on the rise globally for the past two decades, including in the United States, despite a decline in the incidence of certain other cancer forms [1]. Thyroid cancer is three times more prevalent in women than in men. The types of thyroid cancer are papillary carcinoma, follicular carcinoma, Hürthle (oncocytic) cell carcinoma, poorly differentiated carcinoma, medullary carcinoma and anaplastic (undifferentiated) carcinoma [2]. Papillary thyroid carcinoma (PTC) is the most common type of thyroid carcinoma, which accounts for 70% to 80% of all thyroid malignancies. The prognosis of PTC is better than other types of thyroid carcinoma [3]. Thyroid fine needle aspiration (FNA) is an important, safe tool for diagnosing PTC with an accuracy of approximately 94%, and a high degree of sensitivity, specificity [4,5]. Thyroid FNA is applied to distinguish benign from neoplastic or malignant thyroid nodules [6]. The wide use of thyroid FNA has greatly reduced the unnecessary thyroid surgical intervention and thus increased the percent of malignant nodules among all nodules surgically removed. The Bethesda System for Reporting Thyroid Cytopathology (TBSRTC) [7] is the universally accepted reporting system for thyroid FNA diagnosis. Although the cytologic feature of PTC is well documented, including enlarged overlapping nuclei, irregular nuclear contours, intranuclear pseudoinclusions, nuclear grooving, and fine, pale chromatin [8,9], traditionally a time-consuming cytologic analysis is performed by an experienced pathologist who manually examines the glass slides under a light microscope. The most common stain for cytological preparations is the Papanicolaou stain. May-Grünwald Giemsa Stain is one of the common Romanwsky stains used in cytology. Digital pathology has emerged as a potential new standard of care, in which glass slides are digitized into whole slide images (WSIs) using digital slide scanners. With over 100 million pixels in a typical WSI, pathologists find it difficult to manually identify all the information in histopathological images [10]. Thus, an automated diagnosis methods based on artificial intelligence are developed to overcome the constraints of manual and complex diagnosis process [11,12]. In recent years, deep learning has emerged as a potential approach for the automated analysis of medical images. Automating the diagnostic process helps the pathologists to make correct diagnosis in a short period of time. Deep learning has been commonly used to identify diseases such as retinal disease [13], skin cancer [14], colorectal polyp [15], cardiac arrhythmia [16], neurological problems [17], psychiatric problems [18], acute intracranial hemorrhage [19], and autism [20]. Deep learning has also shown the ability to help pathologists diagnose, classify, and segment cancer. For example, Courtiol et al. [21] trained a deep convolutional neural network (MesoNet) to automatically and accurately estimate the overall survival in mesothelioma patients from diagnostic unannotated histopathology images. Yamamoto et al. [22] trained a deep learning based framework that can derive explainable features from diagnostic unannotated histopathology images and anticipate predictions more accurately than humans. Zhang et al. [23] proposed a deep learning-based framework for automating the human-like diagnostic reasoning process, which would include second opinions and thereby encourage clinic consensus. Sanyal et al. [24] trained a convolutional neural network to classify PTC and non PTC on microphotographs from thyroid fine needle aspiration cytology (FNAC). In comparison, Sanyal et al.’s method [24] obtains the diagnostic accuracy of 85.06% on microphotographs of size 512 × 512 pixels from thyroid FNAC while the proposed method achieves an accuracy of 99% on gigapixels WSI of papanicolaou-stained thyroid FNA and ThinPrep (TP) cytological slides for detection and segmentation of PTC. Furthermore, Sanyal et al.’s method [24] can only operate on FNA slides while the proposed method performs consistently well on both thyroid FNA and TP slides. Ke et al. [25] trained a deep convolutional neural network (Faster R-CNN [26]) for detection of PTC from ultrasonic images. To the best of the authors’ knowledge, this is the first study to build an automated deep learning framework for detection and segmentation of PTC from papanicolaou-stained thyroid FNA and TP cytological slides. Figure 1 presents the proposed framework structure and the dataset information. Figure 1a presents the workflow of the system from collection of data to analysis of outcome. Figure 1(ai) shows the thyroid smears are obtained through FNA and TP; in Figure 1(aii), slides of thyroid FNA and TP are prepared with papanicolaou’s staining; in Figure 1(aiii), stained slides are digitalized at 20× objective magnification using Leica AT Turbo scanner; in Figure 1(aiv), digitized whole slide gigapixel images are distributed into a separate training (21%) set and a testing (79%) set; in Figure 1(av), WSIs are processed with fast background filtering of the proposed system; in Figure 1(avi), cytological samples of PTC of individual WSIs are rapidly identified by the proposed deep learning model in seconds. Figure 1b presents the distribution thyroid FNA and TP cytological slides for training and testing. Figure 1c presents the distribution of the number of tiles per WSI. Figure 1d presents the size distribution of the WSIs w.r.t. the width and height. In evaluation, as this is the first study on automatic segmentation of PTC in papanicolaou-stained thyroid FNA and TP cytological slides, we compare the proposed method with the two state-of-the-art deep learning models, including U-Net [27] and SegNet [28].

## 2. Materials and Methods

### 2.1. The Dataset

De-identified and digitized 131 WSIs, including 120 PTC cytologic slides (smear, papanicolaou-stained, *n* = 120) and 11 PTC cytologic slides (TP, papanicolaou-stained, *n* = 11) were obtained from the Department of Pathology, Tri-Service General Hospital, Taipei, Taiwan. All papillary thyroid carcinoma smears were cytologically diagnosed and histologically confirmed by the two expert pathologists. The well-preserved thyroid FNAs, which were done within the last two years, are selected. Ethical approvals have been obtained from the research ethics committee of the Tri-Service General Hospital (TSGHIRB No.1-107-05-171 and No.B202005070), and the data were de-identified and used for a retrospective study without impacting patient care. All the stained slides were scanned using Leica AT Turbo (Leica, Germany), at 20× objective magnification. The average slide dimensions are 77,338 × 37,285 pixels with physical size 51.13 × 23.21 ${\mathrm{mm}}^{2}$. The ground truth annotations were produced by two expert pathologists. The training model uses a total of 28 papanicolaou-stained WSIs (21%), including 25 thyroid FNA and 3 TP cytologic slides. The remaining 103 papanicolaou-stained WISs (79%), including 95 thyroid FNA and 8 TP cytologic slides, are used as a separate testing set for evaluation. The detailed information on the distribution of data could be found in Figure 1b.

### 2.2. Methods

**Figure 2:**
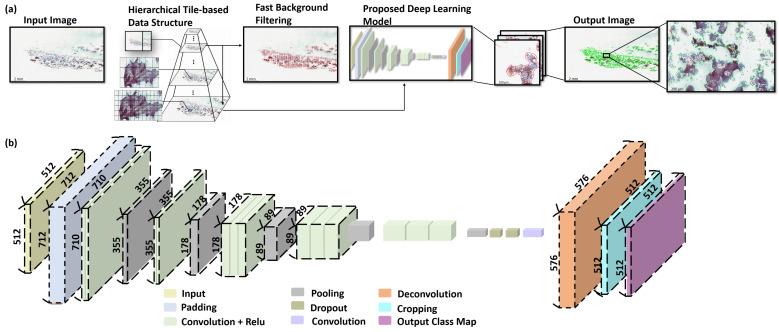
The proposed deep learning framework for segmentation of PTC from papanicolaou-stained thyroid FNA and TP WSIs. (**a**) The workflow of the proposed deep learning framework. Initially, each WSI is formatted into hierarchical data structure and processed by fast background filtering and the proposed deep learning model is used to produce the segmentation results of PTC on papanicolaou-stained thyroid FNA and TP WSIs. (**b**) The detailed architecture of the proposed deep learning model.

In this work, we propose a fast and efficient deep learning based framework for segmentation of PTC in papanicolaou-stained thyroid FNA and TP cytological slides. Figure 2a presents the workflow of the proposed framework. Initially, each WSI is formatted into hierarchical tile-based data structure and assessed by the proposed deep learning model to produce the segmentation results of PTC in papanicolaou-stained thyroid FNA and TP WSIs. Figure 2b shows the detailed architecture of the proposed deep learning model.

#### 2.2.1. Whole Slide Image Processing

Figure 2a presents the framework for segmentation of PTC from papanicolaou-stained thyroid FNA and TP WSIs. Initially, each WSI is formatted into hierarchical data structure and processed by fast background filtering to efficiently discard all the background and reduce the amount of computation per slide and the proposed deep learning model is used to produce the segmentation results of PTC on papanicolaou-stained thyroid FNA and TP WSIs. The details of the proposed WSI processing framework is described in Section S1.1 of the Supplementary Methods and Evaluation Metrics.

#### 2.2.2. Proposed Convolution Network Architecture

The proposed deep learning network is built using VGG16 model as a backbone and adapted from a fully convolutional network framework [29], which has been widely employed in the field of pathology such as neuropathology [30], histopathology [31], and microscopy [32]. The proposed deep learning network consists of a padding layer, six convolutional layers, five max-pooling layers, two dropout layers, one deconvolutional layer, and a cropping layer (see Section S1.2 in Supplementary Methods and Evaluation Metrics for details). The detailed architecture of the proposed deep learning network is shown in Table 1 and Figure 2c.

#### 2.2.3. Implementation details

The proposed method uses the VGG16 model as the backbone for training, with the network optimized using stochastic gradient descent (SGD) optimization and the cross entropy function as a loss function. Furthermore, the network training parameters of the proposed method, including the learning rate, dropout ratio, and weight decay, are set as $1\times 10^{-10}$, 0.5, and 0.0005, respectively. The benchmark methods (U-Net and SegNet) are implemented using the keras implementation [33]. For training, the benchmark methods (U-Net and SegNet) are initialized using a pre-trained VGG16 model, and the network is optimized using Ada-delta optimization with the cross entropy function as a loss function. Furthermore, the network training parameters of the benchmark methods, including the learning rate, dropout ratio, and the weight decay are set to 0.0001, 0.2, and 0.0002, respectively. The proposed method and the benchmark methods (U-Net and SegNet) uses the same framework to process a WSI, which is described in Section 2.2.

## 3. Results

### 3.1. Evaluation Metrics

The quantitative evaluation is produced using five measurements, i.e., accuracy, precision, recall, F1-score, and Jaccard-Index. The evaluation metrics are described in Section S2 of Supplementary Methods and Evaluation Metrics.

### 3.2. Quantitative Evaluation with Statistical Analysis

**Figure 3:**
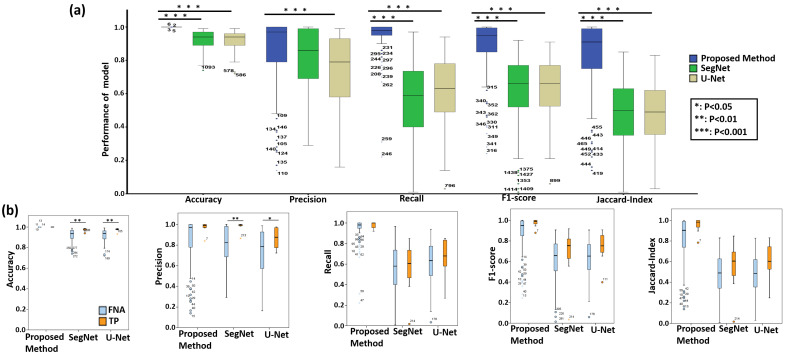
(**a**) The box plot of quantitative evaluation results of the proposed method and the benchmark methods for PTC segmentation. The results of LSD tests shows that the proposed method significantly outperforms the benchmark methods in terms of accuracy, recall, F1-score, and Jaccard-Index (p<0.001). (**b**) The box plot of quantitative evaluation results of the proposed method and the benchmark methods for PTC segmentation from papanicolaou-stained thyroid FNA and TP cytological slides. The results shows that the proposed deep learning framework performs consistently well for the segmentation of PTC on papanicolaou-stained thyroid FNA and TP WSIs and the cell types of thyroid FNA and TP cytological slides do not affect the judgment of the proposed deep learning model while the benchmark methods perform inconsistent for the segmentation of PTC on papanicolaou-stained thyroid FNA and TP WSIs. The box plot of quantitative evaluation results of PTC segmentation where the outliers >1.5× interquartile range are marked with a dot and the outlier >3× interquartile are marked with an asterisk.

The aim of this study is to develop a deep learning framework that can automatically detect PTC from both papanicolaou-stained thyroid FNA and TP cytological slides. For quantitative evaluation, we compared the performance of the proposed method with the state-of-the-art deep learning models, including U-Net and SegNet. Table 2 shows the quantitative evaluation results for segmentation of PTC from papanicolaou-stained thyroid FNA and TP cytological slides. The experimental results show that overall the proposed method achieves the highest accuracy 99%, precision 86%, recall 94%, F1-score 88% and Jaccard 82%, and outperforms the two benchmark approaches. For TP slides, the proposed method obtains even better results with accuracy 99%, precision 97%, recall 98%, F1-score 98% and Jaccard 96%. Figure 3 presents the box plots of the quantitative evaluation results, showing that (a) the proposed method works constantly well overall and significantly outperforms the benchmark methods in terms of accuracy, recall, F1-score, and Jaccard-Index (p<0.001) and (b) the type of cytological slides, i.e., FNA or TP, does not affect the performance of the proposed model, which consistently performs well for both kinds of data while the benchmark approaches tend to perform better in TP than FNA w.r.t. accuracy and precision (p<0.05).

**Figure 4:**
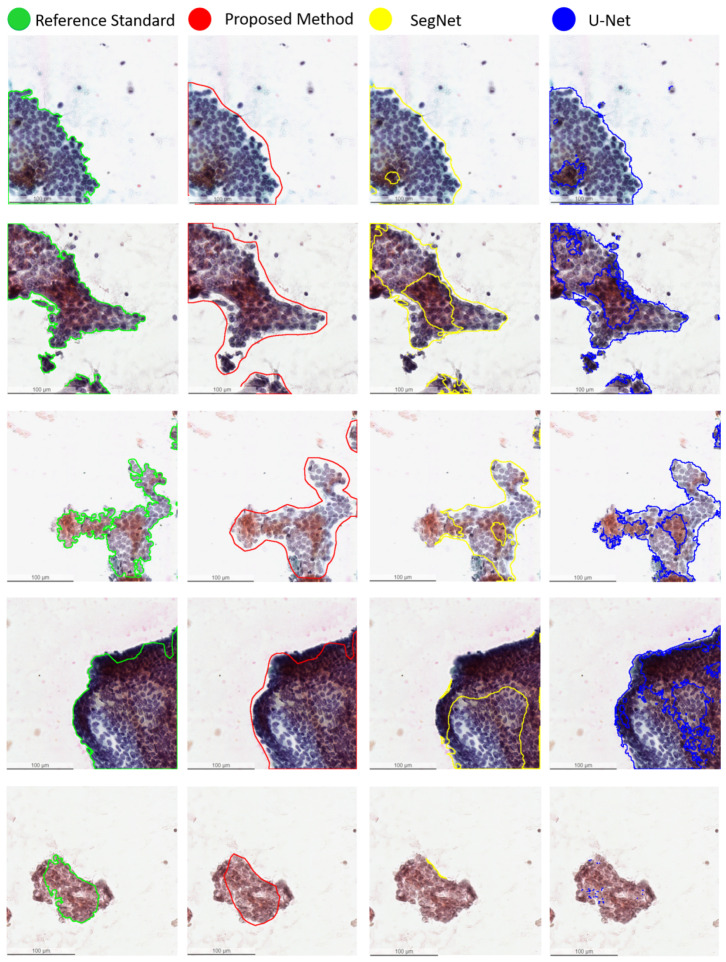
Qualitative segmentation results of the proposed method and two benchmark methods (U-Net and SegNet) for segmentation of PTC on papanicolaou-stained thyroid FNA and TP WSIs.

**Figure 5:**
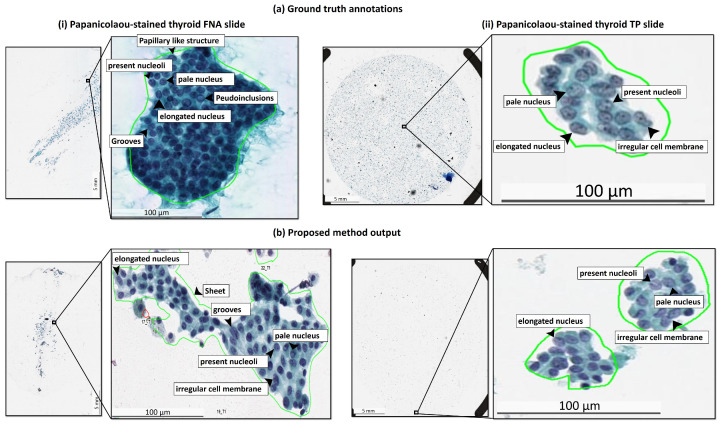
Typical PTC image features on manual annotations by the expert pathologists and automatic segmentation results by the proposed method. (**a**) Annotations by the expert pathologists on FNA and TP WSIs with typical PTC features, including papillary structure, enlarged nucleus, pale nucleus, pseudoinclusions in nuclear cytoplasm, nucleoli and longitudinal grooves and irregular cell membranes. (**b**) Automatic segmentation results by the proposed method with typical PTC features.

For statistical analysis, the quantitative scores were analyzed with the Fisher’s Least Significant Difference (LSD) test using SPSS software (see Table 3). In comparison, the proposed method significantly outperforms the benchmark methods (U-Net and SegNet) in terms of accuracy, recall, F1-score, and Jaccard-Index, based on LSD test (p<0.001). The experimental results demonstrate the high accuracy, efficiency, and reliability of the proposed method on papanicolaou-stained thyroid FNA and TP cytological slides. Figure 4 compares the qualitative segmentation results of the proposed method and two benchmark methods (U-Net and SegNet) in FNA and TP WSIs, showing that the proposed method is able to segment PTC from papanicolaou-stained thyroid FNA and TP slides consistent with the reference standard while the state-of-the-art benchmark methods (U-Net and SegNet) are unable to detect the PTC in some cases. Figure 5 further shows the annotations produced by the expert pathologists with typical PTC features, including papillary like structure, elongated nucleus, pale nucleus, pseudoinclusions in nuclear cytoplasm, nucleoli and longitudinal grooves in FNA and TP slides and the results by the proposed method with typical PTC features as well.

### 3.3. Run Time Analysis

Due to enormous size of WSI, the computing time of WSI analysis is crucial for practical clinical use. We examined the computing time using various hardware configurations (see Table 4). Table 4 compares the computational efficiency of the proposed method with the benchmark methods (U-Net and SegNet), showing that the proposed method takes 0.4 minute to process a WSI using four GeForce GTX 1080 Ti GPUs and 1.7 minute using a single GeForce GTX 1080 Ti GPU, whereas the U-Net model takes 13.2 minutes and the SegNet model takes 15.4 minutes. In addition, even with a single low-cost GPU, the proposed method outperforms the benchmark approaches with less computing time and is 7.8 times faster than U-Net and 9.1 times faster than SegNet. Overall, the proposed method is shown to be capable of detecting PTC reliably in both FNA and TP WSIs and rapidly in seconds, making it suitable for practical clinical use.

## 4. Discussion

In this study, we present a fully automatic and efficient deep learning framework for segmentation of PTC from both papanicolaou-stained thyroid FNA and TP cytological slides. PTC is the most common form of the thyroid cancer with best prognosis and most patients can be cured if treated appropriately and early enough. Thyroid FNA, in addition to pathological examination, is considered the most effective approach for the clinical diagnosis of PTC due to its diagnostic safety, minimal invasiveness and high accuracy. Manual pathological diagnosis is sometimes difficult, time-consuming, and laborious task. In cytopathological diagnosis, pathologists have to conduct a thorough inspection of all information on the glass slides under a light microscope. In recent years, digital pathology in which glass slides are converted into WSIs, has emerged as a potential new standard of care, allowing pathological images to be examined using computer-based algorithms. A typical WSI comprises of more than 100 million pixels, which makes it difficult for pathologists to manually conduct a thorough inspection of all information on cytopathological and histopathological slides. Algorithm-assisted pathologists on WSI diagnosis revealed higher accuracy than either the algorithm or the pathologist alone on review of lymph nodes for metastatic breast cancer, especially improved the sensitivity of detection for micrometastases (91% vs. 83%, *p* = 0.02) [34]. Pathologists easily over-calculate the percent of tumor cells, and the use of AI-based analysis increases the accuracy in applying tumor cell count to genetic analysis [35]. Although artificial intelligence has the potential to provide advantages in accuracy, precision, and efficiency through the automation of digital pathology. Artificial intelligence-related applications are also facing challenges, including regulatory roadblocks, quality of data, interpretability, algorithm validation, reimbursement, and clinical adoption [36]. There are compelling reasons to believe that digital pathology in addition to artificial intelligence for cytological PTC diagnosis is a viable answer to this problem because it aids in the production of more accurate diagnoses, shortens examination times, reduces the pathologists’ workload, and lowers examination cost. For diagnosis of WSIs, many current algorithms are not well adapted to clinical applications due to the high computational cost of employing computational techniques. For practical clinical usage, we develop a fast, efficient, and fully automatic deep learning framework for fast screening of both FNA and TP slides. The experimental results show that the proposed deep learning framework has been demonstrated to be effective, as the proposed method achieves the accuracy and recall of over 90%. Furthermore, the proposed method achieves significantly superior performance than the state-of-the art deep learning models, including U-Net and SegNet using Fisher’s LSD test (p<0.001). The results demonstrate that the proposed method is able to segment PTC with high accuracy, precision, and sensitivity, comparable to the referenced standard produced by pathologists in seconds laying the groundwork for the use of computational decision support systems in clinical practice. The including 131 cytologic slides are all definitive papillary thyroid carcinoma under TBSRTC criteria. The limitation of our study is that in practice, cytopathologists have to face quantitatively insufficient specimens for a definitive diagnosis, for instance, the diagnosis of “suspicious for PTC”. In future work, the proposed framework could be extended to the detection and segmentation of “suspicious for PTC” slides and different types of carcinoma, such as follicular carcinoma, Hürthle (oncocytic) cell carcinoma, medullary carcinoma, poorly differentiated carcinoma and anaplastic carcinoma.

## 5. Conclusions

In this work, we introduce a deep learning-based framework for automatic detection and segmentation of PTC in papanicolaou-stained thyroid FNA and TP cytological slides. We evaluated the proposed framework on a dataset of 131 WSIs, including 120 PTC cytologic slides (smear, papanicolaou-stained, *n* = 120) and 11 PTC cytologic slides (TP, papanicolaou-stained, *n* = 11), and the experimental results show that the proposed method achieves high accuracy, precision, recall, F1-score, and Jaccard-Index. In addition, we compared the proposed method with the state-of-the-art deep learning models, including U-Net and SegNet. Based on Fisher’s LSD test, the proposed method significantly outperforms the two benchmark methods in terms of accuracy, recall, F1-score, and Jaccard-Index (p<0.001). 

## Figures and Tables

**Table 1:** The architecture of the proposed deep learning model.

Layer	Features (Train)	Features (Inference)	Kernel Size	Stride
Input	512 × 512 × 3	512 × 512 × 3	-	-
Padding	712 × 712 × 3	712 × 712 × 3	-	-
Conv1_1 + relu1_1	710 × 710 × 64	710 × 710 × 64	3 × 3	1
Conv1_2 + relu1_2	710 × 710 × 64	710 × 710 × 64	3 × 3	1
Pool1	355 × 355 × 64	355 × 355 × 64	2 × 2	2
Conv2_1 + relu2_1	355 × 355 × 128	355 × 355 × 128	3 × 3	1
Conv2_2 + relu2_2	355 × 355 × 128	355 × 355 × 128	3 × 3	1
Pool2	178 × 178 × 128	178 × 178 × 128	2 × 2	2
Conv3_1 + relu3_1	178 × 178 × 256	178 × 178 × 256	3 × 3	1
Conv3_2 + relu3_2	178 × 178 × 256	178 × 178 × 256	3 × 3	1
Conv3_3 + relu3_3	178 × 178 × 256	178 × 178 × 256	3 × 3	1
Pool3	89 × 89 × 256	89 × 89 × 256	2 × 2	2
Conv4_1 + relu4_1	89 × 89 × 512	89 × 89 × 512	3 × 3	1
Conv4_2 + relu4_2	89 × 89 × 512	89 × 89 × 512	3 × 3	1
Conv4_3 + relu4_3	89 × 89 × 512	89 × 89 × 512	3 × 3	1
Pool4	45 × 45 × 512	45 × 45 × 512	2 × 2	2
Conv5_1 + relu5_1	45 × 45 × 512	45 × 45 × 512	3 × 3	1
Conv5_2 + relu5_2	45 × 45 × 512	45 × 45 × 512	3 × 3	1
Conv5_3 + relu5_3	45 × 45 × 512	45 × 45 × 512	3 × 3	1
Pool5	23 × 23 × 512	23 × 23 × 512	2 × 2	2
Conv6 + relu6 + Drop6	17 × 17 × 4096	17 × 17 × 4096	7 × 7	1
Conv7 + relu7 + Drop7	17 × 17 × 4096	17 × 17 × 4096	1 × 1	1
Conv8	17 × 17 × 3	17 × 17 × 3	1 × 1	1
Deconv9	576 × 576 × 3	576 × 576 × 3	64 × 64	32
Cropping	512 × 512 × 3	512 × 512 × 3	-	-
Output Class Map	512 × 512 × 1	512 × 512 × 1	-	-

**Table 2:** Quantitative evaluation for segmentation of PTC in thyroid FNA and TP slides.

	Proposed Method			U-Net [27]			SegNet [28]		
	All	FNA	TP	All	FNA	TP	All	FNA	TP
Accuracy	**0.99**	**0.99**	**0.99**	0.92	0.92	0.98	0.92	0.92	0.97
Precision	**0.86**	**0.85**	0.97	0.74	0.73	0.87	0.81	0.80	**0.98**
Recall	**0.94**	**0.94**	**0.98**	0.61	0.61	0.66	0.56	0.56	0.55
F1-score	**0.88**	**0.87**	**0.98**	0.64	0.63	0.74	0.62	0.61	0.67
Jaccard-Index	**0.82**	**0.80**	**0.96**	0.49	0.48	0.60	0.48	0.47	0.54

**Table 3:** Statistical analysis: multiple comparisons for segmentation of PTC.

Measurement	(I) Method	(J) Method	Mean Diff. (I–J)	Std. Error	Sig.	95% C.I.	
						Lo. Bound	Up. Bound
Accuracy	Proposed Method	U-Net	0.0784732 *	0.0068	<0.001	0.0651	0.0918
		SegNet	0.0761947 *	0.0068	<0.001	0.0629	0.0895
Precision	Proposed Method	U-Net	0.1187516 *	0.0289	<0.001	0.0620	0.1755
		SegNet	0.0452	0.0289	0.1180	−0.0116	0.1020
Recall	Proposed Method	U-Net	0.3336451 *	0.0271	<0.001	0.2803	0.3870
		SegNet	0.3856975 *	0.0271	<0.001	0.3323	0.4391
F1-score	Proposed Method	U-Net	0.2392282 *	0.0266	<0.001	0.1868	0.2916
		SegNet	0.2578479 *	0.0266	<0.001	0.2055	0.3102
Jaccard Index	Proposed Method	U-Net	0.3238887 *	0.0290	<0.001	0.2668	0.3809
		SegNet	0.3391651 *	0.0290	<0.001	0.2821	0.3962

* The proposed method is significantly better than the benchmark methods (U-net [27] and SegNet [28]) (*p* < 0.001).

**Table 4:** Comparison on hardware and computing time per WSI.

Method	CPU	RAM	GPU	Time (min) *
Proposed Method	Intel Xeon Gold 6134 CPU @ 3.20GHz × 16	128 GB	4 × GeForce GTX 1080 Ti	**0.4**
Proposed Method	Intel Xeon CPU E5-2650 v2 @ 2.60GHz × 16	32 GB	1 × GeForce GTX 1080 Ti	1.7
U-Net [27]	Intel Xeon CPU E5-2650 v2 @ 2.60GHz × 16	32 GB	1 × GeForce GTX 1080 Ti	13.2
SegNet [28]	Intel Xeon CPU E5-2650 v2 @ 2.60GHz × 16	32 GB	1 × GeForce GTX 1080 Ti	15.4

* The size of WSI in this evaluation is 4,069,926,912 pixels (91,632 × 44,416 pixels).

## Data Availability

The data that support the findings of this study are available from the corresponding author upon reasonable request.

## Supplementary Materials

The following are available at https://www.mdpi.com/article/10.3390/cancers13153891/s1, Supplementary Methods and Evaluation Metrics.
