# Supplementary material for: Deep Learning Fast Screening Approach on Cytological Whole Slides for Thyroid Cancer Diagnosis
Source: Cancers (Basel). 2021 Aug 2;13(15):3891. doi: 10.3390/cancers13153891 (PMC8345428; doi:10.3390/cancers13153891)
Supplement: Supplementary file 1 [file cancers-13-03891-s001.zip › cancers-1293334-supplementary.pdf]

Supplementary Methods and Evaluation Metrics

1 Methods

1.1 Whole Slide Image Processing

To deal with gigapixel efficiently, each WSI $W(a,b)$ is formatted as tile based data structure $\mathcal{T} = \{t^{u}_{\rho,\sigma}(i,j)\}^{N}_{u=1} \in W(a,b)$, where ρ , σ , i , j represents the patch column index, patch row index, patch horizontal sub-index, patch vertical sub-index and the image level, respectively. When u = N , ρ , σ , i , j are calculated as follows:

$$\begin{aligned} \rho &= \lfloor a/\zeta_\theta \rfloor, \qquad \sigma = \lfloor b/\eta_\theta \rfloor, \\ i &= a - \rho \times \zeta_\theta, \quad j = b - \sigma \times \eta_\theta \end{aligned} \tag{1}$$

where ζ_θ , ψ_θ denote the patch width and patch height, respectively. The values ρ , σ , i , j are in range $\{0, \cdots, \alpha-1\}$, $\{0, \cdots, \beta-1\}$, $\{0, \cdots, \zeta_\theta - 1\}$ and $\{0, \cdots, \eta_\theta - 1\}$, respectively; ($\zeta_\theta, \eta_\theta$) = (512,512) in this study.

Initially, to efficiently discard all the background and reduce the amount of computation per slide, each WSI is processed with fast background filtering by Otsu applied onto $t^{x}_{\rho,\sigma}(i,j)$; at the level closet to a patch size. The value of x is calculated as follows:

$$z = argmin_u(\alpha \times \beta > 1 \wedge card(t^u) \geq \zeta_\theta \times \eta_\theta) \tag{2}$$

Then, for fast WSI analysis, the proposed deep learning model D is applied, which is discussed in section 1.2. Each tile of $t^{N}_{\rho,\sigma}(i,j)$ is processed by the proposed deep learning model D to get the probabilities of cancer cells as shown in eq.(3).

$$p^{N}_{\rho,\sigma}(i,j)^l = D(t^{N}_{\rho,\sigma}(i,j)) \tag{3}$$

where $l = 0, \cdots, L$ represents number of types of tissue to be identified, and 0, 1 and 2 represent the background, the non-target and the target tissue type, respectively.

Next, a two dimensional pixel-based class map is obtained as the index of the tissue type that has the maximum probability of the pixel using eq.(4).

$$c^{N}_{\rho,\sigma}(i,j) = argmax_l((p^{N}_{\rho,\sigma}(i,j)^l)) \tag{4}$$

Then, the pixel-based segmentation result of cancer cells $\mathcal{S} = \{s^{N}_{\rho,\sigma}(i,j)\}$ are obtained based on class map $c^{N}_{\rho,\sigma}(i,j)$ using eq.(5).

$$s^{N}_{\rho,\sigma}(i,j) = \begin{cases} W_{\rho,\sigma}(i,j) & , c^{N}_{\rho,\sigma}(i,j) > 1 \\ \phi & , otherwise \end{cases} \tag{5}$$

1.2 Proposed Convolution Network Architecture

The proposed deep learning model includes a padding layer, six convolutional layers, five max-pooling layers, two dropout layers, one deconvolutional layer, and a cropping layer. Firstly, padding is applied to the input image in order to increase the size of input image from 512 × 512 × 3 to 712 × 712 × 3. After padding, five convolutional layers are applied, including the first two layers for two convolution sequences with a kernel size of 3 × 3 and stride size of 1 and the last three convolutional layers for three convolution sequences with a kernel size of 3 × 3 and stride size of 1, and ReLU is applied after each convolution layer. The output dimensions of each convolution layer are formulated as follows.

$$(f_h, f_w, f_r) = (\lfloor \frac{q_h + 2e - k}{o} + 1 \rfloor, \lfloor \frac{q_w + 2e - k}{o} + 1 \rfloor, q_k) \tag{6}$$

where q_h and q_w are the height and width of the input, respectively; e is the padding size; k is the kernel size; o is the stride size; q_k is the number of filters used; f_h , f_w , and f_r are the height, width and number of channels of the output of each convolution layer, respectively.

Each convolution is followed by a max pooling layer with a kernel size of 2 × 2 and stride size of 2, to down sample the feature maps. The output dimensions of each pooling layer are formulated as follows.

$$(y_h, y_w, y_r) = (\lceil \frac{q_h + 2e - k}{o} + 1 \rceil, \lceil \frac{q_w + 2e - k}{o} + 1 \rceil, q_c) \tag{7}$$

where q_c is the number of channels of the input; y_h , y_w , and y_r are the height, width, and number of channels of the output pooling layer, respectively.

After five convolution layers and five max pooling layers, there are two drop out layers (dropout ratio of 0.5) followed by two convolutions with a kernel size of 7 × 7 and stride size of 1 for first convolution, and a kernel size of 1 × 1 and stride size of 1 for second convolution, respectively, and ReLU is applied after each convolution. After two drop out layers, there is a convolution layer with a kernel size of 1 × 1 and a stride size of 1 to decrease the number of output channels. Following the convolution layer, there is a deconvolution layer with kernel size of 64 × 64 and stride size of 32 , which is used to restore the feature maps to the same size as the padding image, and hence a prediction could be generated for each pixel while retaining the spatial information in the original image and on the upsampled feature map. Following deconvolution layer, cropping is done in order to match with the input size. After cropping, a two dimensional pixel-based class map is produced as the index of the tissue type that has the highest probability of the pixel using section 1.1 eq.(4).

2 Evaluation Metrics

Quantitative evaluation on the segmentation performance is produced using five measurements, i.e. Accuracy, Precision, Recall, F1-score, and Jaccard-Index. The evaluation metrics are computed as follows.

$$Accuracy = \frac{TP + TN}{TP + TN + FP + FN} \tag{8}$$

$$Precision = \frac{TP}{TP + FP} \tag{9}$$

$$Recall = \frac{TP}{TP + FN} \tag{10}$$

$$F1 - score = \frac{2TP}{2TP + FP + FN} \tag{11}$$

$$Jaccard - Index = \frac{F1 - score}{2 - F1 - score} = \frac{TP}{TP + FN + FP} \tag{12}$$

where TP denotes the true positive, TN represents the true negative, FP denotes false positive, and FN is the false negative.
